# Integration of miRNA profiles and clinical data for early risk assessment of bronchopulmonary dysplasia in VLBW and ELBW newborn infants: a discovery study

**DOI:** 10.3389/fped.2026.1853322

**Published:** 2026-07-06

**Authors:** Iskander Isgandarov, Arailym Abilbayeva, Anel Tarabayeva, Dinara Yelyubayeva, Nishankul Bozhbanbayeva, Ingkar Okhas, Nuray Shaktay, Dana Yerbolat, Kristina Kovaleva, Zhanar Akhmetova, Zulfiya Kachiyeva, Aibek Smagul

**Affiliations:** 1 Institute of Plant Biology and Biotechnology, Almaty, Kazakhstan; 2 Shortanbayev General Immunology Department, Asfendiyarov Kazakh National Medical University, Almaty, Kazakhstan; 3 Department of Highly Dependent Newborns, The Center for Perinatology and Pediatric Cardiac Surgery, Almaty, Kazakhstan; 4 Neonatology Department, Asfendiyarov Kazakh National Medical University, Almaty, Kazakhstan; 5 Department of Biophysics, Biomedicine and Neuroscience, Al-Farabi Kazakh National University, Almaty, Kazakhstan; 6 B. Atchabarov Scientific- Research Institute of Fundamental and Applied Medicine, Asfendiyarov Kazakh National Medical University, Almaty, Kazakhstan; 7 Genome Centre, Asfendiyarov Kazakh National Medical University, Almaty, Kazakhstan; 8 Department of Natural Sciences Pedagogy, School of Education and Humanities, SDU University, Kaskelen, Kazakhstan

**Keywords:** biomarkers, bronchopulmonary dysplasia, discovery study, miRNA, predictive modelling, preterm infants, risk prediction

## Abstract

**Background and objectives:**

Bronchopulmonary dysplasia (BPD) remains a significant complication linked to prematurity, especially in infants with very and extremely low birth weights. A major challenge in identifying prognostic biomarkers is the biological noise in blood samples, which masks the specific signals of BPD. This study aimed to identify candidate miRNA markers in the blood and develop integrated models for early BPD risk assessment.

**Materials and methods:**

A prospective cohort study was conducted on preterm infants with birth weights below 1,500 g. Among the 41 infants in the cohort, 20 developed BPD and 21 did not. Peripheral blood samples were collected at 10–14 days of life, after which samples were analyzed using GeneChip™ miRNA 4.1. Differential expression was determined using limma, with a false discovery rate threshold of 0.05. To identify candidate miRNAs associated with systemic inflammatory markers and exclude non-specific signals from modeling, Spearman's correlation analysis was subsequently performed. Next, a predictive model was developed using LASSO regression and validated through leave-one-out cross-validation. All statistical analyses were performed using R.

**Results:**

miRNA expression analysis identified 5 molecules with significantly higher levels in BPD (adjusted *p* < 0.05). Spearman correlation analysis revealed no significant associations between the five candidate miRNAs and systemic inflammatory parameters. Four miRNAs were selected based on the highest composite evidence scores across statistical significance, discriminative performance, and penalized regression stability criteria, comprising hsa-let-7b-5p, hsa-miR-27a-3p, hsa-let-7c-5p, and hsa-miR-182-5p. The final model, integrating clinical data and miRNA predictors, achieved an adjusted LOOCV-AUC of 0.940 (95% CI 0.861–1.000), outperforming the model with only clinical data (AUC 0.719, 95% CI 0.557–0.868).

**Conclusion:**

A combined model based on miRNA and clinical data showed promising results for early assessment of bronchopulmonary dysplasia risk, achieving a cross-validated AUC of 0.940 in a small cohort. A correlation-based sensitivity analysis reduced systemic impact, enabling the identification of specific candidate markers. These results require external validation and confirmation by RT-qPCR before clinical implementation.

## 1 Introduction

Bronchopulmonary dysplasia (BPD) represents a significant complication of prematurity, frequently resulting in long-term disability and persistent respiratory problems (1–3). The risk is greatest among newborns with a birth weight below 1,500 g (4). Although respiratory support has advanced, early prediction of BPD remains a major challenge in neonatology. Existing diagnostic criteria confirm the disease retrospectively, relying on oxygen therapy requirements, which limits the ability to individualize treatment strategies at an early stage (5).

The search for reliable prognostic biomarkers currently employs several strategies, including the evaluation of traditional clinical predictors such as anthropometric data and Apgar scores (6, 7), analysis of genetic polymorphisms and proinflammatory cytokine profiles (8–10), and examination of miRNA expression patterns as post-transcriptional regulators of lung development (11, 12). Traditional clinical predictors demonstrate limited specificity, genetic status reflects only predisposition, and cytokine and matrix metalloproteinase levels exhibit considerable fluctuation with low specificity. Although specific miRNA families, such as the miR-17–92 cluster, have been implicated in the pathogenesis of bronchopulmonary dysplasia (11), no validated miRNA-based diagnostic tool is currently available for clinical application. This underscores the necessity for systematic approaches to biomarker discovery.

In this context, research on miRNAs that regulate post-transcriptional gene expression is receiving increasing attention in neonatology (12–15). Circulating miRNAs can rapidly indicate changes in cellular homeostasis and have shown promise as biomarkers for critical neonatal conditions, including BPD (16). However, their clinical implementation encounters significant challenges. The heterogeneous clinical status of premature infants, driven by systemic inflammatory responses, comorbidities, and developmental immaturity, yields a nonspecific molecular profile (17–19). This considerable biological noise complicates the identification of miRNAs specifically associated with the pathogenetic progression of BPD, as opposed to those reflecting general adaptive responses.

A key challenge is selecting the most appropriate biomaterial. Blood is the most accessible and clinically suitable source in premature infants, since obtaining respiratory tract aspirates is traumatic and technically challenging. However, blood serves as a universal transport medium, collecting markers from all injured organs and tissues (20, 21). Given this systemic quality, it is crucial to identify and exclude candidate markers that correlate with nonspecific inflammatory parameters, thereby isolating those specific to bronchopulmonary dysplasia rather than general host responses.

This study introduces a method to mitigate biological noise by employing correlation-based screening of transcriptomic data. The central hypothesis posits that specific miRNAs involved in post-transcriptional regulation of lung development may serve as candidate predictors of BPD, provided that molecules associated with nonspecific systemic responses are excluded.

Study objective: to develop and internally validate an exploratory diagnostic model for early assessment of BPD risk in VLBW and ELBW infants by integrating clinical data with miRNA profiles, while using correlation sensitivity analysis to reduce the potential influence of systemic inflammatory signals.

## 2 Materials and methods

### 2.1 Study design and ethical approval

This prospective cohort study was conducted at the Neonatal Intensive Care Unit (NICU) of the Center for Perinatology and Pediatric Cardiac Surgery in Almaty from January to July 2025. The study protocol was first reviewed and approved by the Local Ethics Committee (Protocol No. 19 (155), dated September 30, 2024). The research strictly followed the ethical principles set out in the World Medical Association Declaration of Helsinki. Informed consent was obtained from the legal guardians of all newborns before any procedures, including consent for participation and data processing.

### 2.2 Criteria for cohort formation

The study focused on premature infants weighing under 1,500 g. It excluded full-term infants, premature infants weighing over 1,500 g, and those with confirmed congenital malformations, genetic abnormalities, or chromosomal disorders.

### 2.3 Sampling procedure and stratification

**Figure 1:**
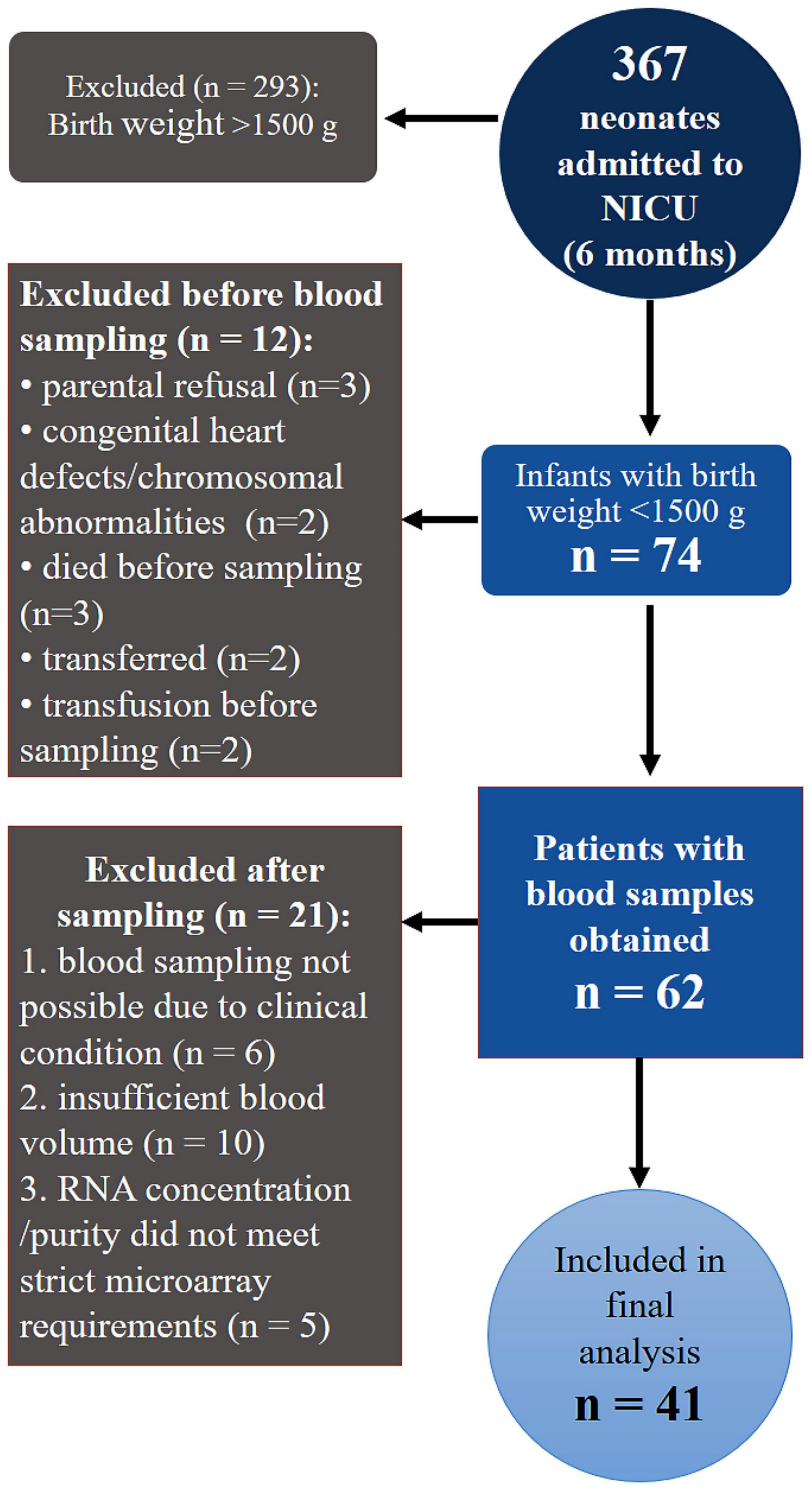
Flowchart of patient selection and study design. A total of 62 preterm infants were screened. Twenty-one were excluded based on predefined criteria. The final cohort comprised 41 infants stratified into two groups: BPD+ (*n* = 20) and BPD− (*n* = 21). Peripheral blood sampling for miRNA profiling was performed on days 10–14 of life.

Peripheral blood sampling was performed between days 10 and 14 of life. The final verification of the BPD diagnosis was performed on the 28th day of life in accordance with the 2018 NIH consensus criteria. The 2018 NIH consensus criteria were applied as the primary diagnostic framework, guided by three considerations. Their operational definition is anchored at day 28 of postnatal life, which directly corresponds to the primary outcome time point of this study. Furthermore, all BPD + infants in the cohort retained respiratory support requirements at 36 weeks postconceptional age, confirming concordance with the Jensen 2019 severity-graded classification (22). Additionally, the 2018 NIH criteria represent the current operational standard within neonatal intensive care protocols in Kazakhstan. From the original cohort of 62 preterm infants, 41 were included in the final analysis. This resulted in two groups: the study group (BPD+, *n* = 20) and the comparison group (BPD−, *n* = 21). The detailed patient selection process and the final group formation stages are shown in the flowchart (Figure 1).

### 2.4 MiRNA expression profiling

For molecular genetic analysis, 1–2 mL of venous blood was collected into K2-EDTA anticoagulated vacuum tubes. The samples were promptly transported under cold chain conditions to the B.A. Atchabarov Center for Collective Use research laboratory, with the temperature kept at or below +4 °C.

#### 2.4.1 Sample processing and storage

In the laboratory, blood samples were initially prepared through aliquoting and fractionation. Following centrifugation at 3,000 rpm for five minutes, serum and plasma were separated. The remaining whole blood was then frozen and stored at −80 °C for subsequent analysis.

#### 2.4.2 Extraction of miRNA from blood

To extract miRNA from whole venous blood, we employed the MagMAX™ mirVana™ Total RNA Isolation Kit (Thermo Fisher Scientific, A27828), specifically crafted for isolating total RNA, including small RNA fractions. We chose this kit because of its versatility, enabling the extraction of miRNA from different biological samples with a single protocol.

RNA was extracted using MagMAX magnetic particle technology in accordance with the manufacturer's instructions. For whole blood samples, the Isolate RNA from Whole Blood Samples protocol was employed. All preparation and extraction steps were performed under RNase-free conditions to prevent nucleic acid degradation. Certified nuclease-free consumables and filter-barrier pipette tips were used throughout the procedure. Work surfaces, pipettes, and gloves were decontaminated with an RNase-inactivating reagent (RNaseZap™) prior to each run, and lysates and eluates were maintained on ice during all manual handling steps.

Following elution, isolated RNA was subjected to quality assessment prior to downstream processing. RNA purity was evaluated spectrophotometrically using a NanoDrop™ One C instrument, with A260/A280 ratios of approximately 2.0 and A260/A230 ratios of 1.8 or above considered acceptable. Given that UV spectrophotometry does not adequately reflect small RNA recovery or integrity, RNA concentration was additionally determined by fluorometric quantification (Qubit™), and the absence of gross degradation was confirmed by conventional agarose gel electrophoresis. Samples that did not meet the predefined purity and integrity criteria were excluded from further processing. Qualifying RNA was stored at −80 °C until use.

The MagMAX™ mirVana™ kit enables phenol-free extraction of total RNA, covering all RNA types such as miRNAs, non-coding RNAs, mRNAs, and ribosomal RNA, and is recognized for producing highly consistent and stable RNA yields (23–25).

#### 2.4.3 Biotin labeling, hybridization, and expression analysis of miRNAs on the GeneChip™ miRNA 4.1 array plate platform

Total RNA, including the low-molecular-weight (LMW) fraction containing miRNAs, was eluted in RNase-free water to preserve RNA integrity throughout sample handling and downstream processing. Profiling was performed on GeneChip™ miRNA 4.1 Array Plate microarrays, which accommodate up to 96 samples simultaneously and provide comprehensive coverage of mature miRNA sequences annotated in the miRBase database, making them particularly suitable for high-throughput small non-coding RNA profiling.RNA labeling was carried out using the FlashTag™ Biotin HSR Labeling Kit according to the manufacturer's instructions. Briefly, 2 µL of RNA Spike Control Oligos were combined with 8 µL of total RNA, and the mixture was subjected to a Poly(A) tailing reaction at 37 °C for 15 min using an ATP mix diluted according to the input RNA type and quantity. Biotinylated signal molecules were subsequently incorporated by adding 5X FlashTag Biotin HSR Ligation Mix and T4 DNA Ligase to the tailed RNA, followed by incubation at 25 °C for 30 min. The reaction was terminated with HSR Stop Solution.

Prior to hybridization, ovens, microarrays, and sample log files were configured in GeneChip™ Command Console software as per the manufacturer's guidelines. A hybridization cocktail was then prepared using the GeneTitan™ Hybridization, Wash, and Stain Kit for miRNA Array Plates together with the GeneChip™ Hybridization Control Kit, incorporating hybridization buffer, formamide, DMSO, and standard controls. The labeled sample was added to this cocktail, denatured at 95 °C for 10 min, and then equilibrated at 48 °C for 3 min to facilitate effective probe binding. The mixture was subsequently centrifuged to remove insoluble particulates, and the clarified supernatant was transferred to the designated wells of the HT Hybridization Tray.

Hybridization, washing, and staining were conducted automatically on the GeneTitan™ instrument in accordance with the manufacturer's recommended protocols and temperature settings, using the dedicated GeneTitan™ Hybridization, Wash, and Stain Kit for miRNA Array Plates to ensure procedural consistency and minimize technical variability. Microarray scanning was performed automatically, generating raw CEL files for downstream analysis.

Quality control was applied at both the labeling and hybridization stages, drawing on RNA Spike Control Oligos as well as the built-in hybridization and spike controls of the GeneChip™ miRNA 4.1 Array platform. Labeling efficiency and overall assay performance were monitored in Expression Console™ software, and only samples meeting predefined quality thresholds were carried forward to downstream analysis.

### 2.5 Bioinformatic analysis

Raw Affymetrix CEL files were imported via the oligo package (v1.74.0) and normalized with the Robust Multi-array Average (RMA) method, yielding log_2_-transformed expression values. Probe sets were annotated using the miRNA-4_1 reference (v20160922) and filtered to include only Homo sapiens miRNAs; those with mean log_2_ intensity ≤5 across the cohort were excluded to minimize technical noise. Quality control at the sample level involved examining intensity distributions and detecting outliers; PCA was conducted and visualized with group-colored confidence ellipses to assess cohort separation. Differential miRNA expression between the BPD and control groups was identified using limma (v3.66.0) (26), with empirical Bayes moderation in a covariate-adjusted linear model that included condition, gestational age, and birth weight. Benjamini–Hochberg FDR correction, and cutoffs of |log_2_FC| ≥ 1 and FDR < 0.05 were applied. To assess whether candidate miRNA expression reflected non-specific systemic inflammatory responses rather than BPD-specific biology, Spearman correlation analysis was performed between the expression levels of all differentially expressed miRNAs and routinely collected inflammatory laboratory parameters, namely total white blood cell count (WBC, ×10⁹/L) and C-reactive protein (CRP, mg/L), measured at two time points: day 7 and day 14 of life. miRNAs demonstrating a statistically significant correlation with any of these parameters (*p* < 0.05) were to be excluded from further modeling as potential non-specific inflammatory signals. Targets of the selected miRNAs were predicted using experimentally validated interactions in the multiMiR package (v1.32.0). Only high-confidence, validated targets that met the predefined filtering criteria were retained, and functional enrichment analysis was performed on this restricted target set using clusterProfiler (v4.18.4). Gene Ontology Biological Process (GO-BP) and KEGG pathway enrichment were tested, with significance assessed at *p* < 0.05 and *q* < 0.20 after Benjamini–Hochberg FDR correction. The results were visualized using dot plots and bar plots.

### 2.6 Statistical analysis

Baseline characteristics were categorized by BPD status and summarized as medians with interquartile ranges (IQR) for continuous variables and as counts with percentages for categorical variables. Comparisons between groups employed the Wilcoxon ranksum test and Fisher's exact test (two-tailed, *α* = 0.05). Missing data patterns were evaluated at both the variable and subject levels using naniar (v1.1.0), and continuous variables were imputed through Multiple Imputation by Chained Equations (mice v3.19.0) with predictive mean matching, five imputations, and a fixed seed. For each significant miRNA, ROC analysis (pROC v1.19.0.1) was used to compute AUC values with 95% confidence intervals. miRNAs with an AUC of at least 0.75 were selected as candidates. A concise miRNA panel was created through a four-step approach: (1) tier assignment—miRNAs with *p* < 0.01 and AUC ≥ 0.85 made up the primary tier; if not, the top three based on *p*-value were selected; (2) penalized regression—LASSO (*α* = 1) and elastic net (*α* = 0.5) models were fitted using glmnet (v4.1-10, lambda.1se); (3) stability selection (27) —LASSO was run on 1,000 bootstrap resamples (including 80% of subjects), with >60% selection frequency indicating stable miRNAs; (4) evidence scoring—aiming to identify at least three top candidates. Individual multivariate associations between each candidate miRNA and BPD were assessed using Firth-penalized logistic regression (logistf) adjusted for available clinical covariates to account for potential separation in small samples. Multicollinearity among clinical predictors was assessed using VIFs (car v3.1-5), and variables with VIF > 5 were excluded. miRNAs retaining nominal significance (*p* < 0.05) in Firth-adjusted models were carried forward; if none survived, the top three by nominal *p*-value from the evidence-scored pool were used. Several multivariable models were developed. A base clinical model, individual combined models, and a full combined model incorporating all candidate miRNAs alongside clinical predictors. Each model used Firth-penalized logistic regression (28, 29). It should be noted that miRNA candidate selection (LASSO, elastic net, and stability selection) was performed on the full dataset prior to LOOCV, which may introduce optimistic bias into the reported cross-validated AUC. This limitation is acknowledged in the Limitations section. Internal validation was performed with leave-one-out cross-validation (caret v7.0-1) using standard logistic regression, and overfitting was assessed by comparing AUC differences between training and LOOCV. Lambda selection for the LASSO-based selection steps used the median lambda.1se across 10 repeated 5-fold cross-validations. Optimal thresholds were identified using Youden's index. Calibration was assessed via the Hosmer–Lemeshow test (ResourceSelection v0.3-6), calibration slope, calibration intercept, and Brier score. Bootstrap optimism correction (Harrell method, *B* = 500 iterations) was applied to correct the apparent AUC for overfitting: optimism = mean(AUC_bootstrap—AUC_original); corrected AUC = apparent AUC—optimism. Bootstrap 95% confidence intervals for all AUC values were computed using the percentile method (*B* = 2,000 iterations). Clinical utility was evaluated through decision curve analysis (30) (dcurves v0.5.1) across threshold probabilities 0–0.6. All analyses were performed in R (v4.5.1), and a two-sided *p*-value <0.05 was deemed statistically significant unless specified otherwise.

## 3 Results

### 3.1 Clinical characteristics and neonatal outcomes of the study cohort

To identify potential covariates, a comparative analysis of the clinical and medical history characteristics of newborns in the study group (*n* = 41) was conducted. The findings revealed significant differences between patients with verified bronchopulmonary dysplasia and the control group. Newborns in the study group exhibited severe morphofunctional immaturity, reflected by notably lower gestational ages of 27.5 [27.0–28.0] weeks compared to 29.0 [28.0–31.0] weeks in the control group (*p* = 0.001). A similar trend was observed in anthropometric data, with the median birth weight in the BPD + group significantly lower at 928.0 [692.5–1,061.5] g vs. 1,425.0 [994.0–1,465.0] g in controls (*p* = 0.001). Additionally, BPD patients showed poorer early postnatal adaptation, indicated by a significant lower Apgar score at 5 min (*p* = 0.020). The main group also had a higher incidence of comorbidities, particularly anemia of prematurity (*p* = 0.002), necrotizing enterocolitis (*p* = 0.003), posthemorrhagic anemia (*p* = 0.003), and DIC syndrome (*p* = 0.004) (Table 1).

**Table 1:** Clinical characteristics of preterm infants with and without bronchopulmonary dysplasia.

Variable	*N*	Overall	Control	BPD	*p*-value
		*N* = 41	*N* = 21	*N* = 20	
Gestational age (GA)	41	28.0 [27.0–30.0]	29.0 [28.0–31.0]	27.5 [27.0–28.0]	0.001
Birth weight	41	994.0 [890.0–1,430.0]	1,425.0 [994.0–1,465.0]	928.0 [692.5–1,061.5]	0.001
Apgar score (1 min)	41	4.0 [3.0–5.0]	5.0 [3.0–6.0]	4.0 [2.0–4.0]	0.056
Apgar score (5 min)	41	6.0 [5.0–7.0]	7.0 [5.0–7.0]	5.0 [4.0–6.0]	0.020
Silverman score (1 min)	41	7.0 [7.0–8.0]	7.0 [6.0–8.0]	8.0 [7.0–8.0]	0.144
Silverman score (5 min)	41	8.0 [8.0–9.0]	8.0 [8.0–9.0]	8.0 [8.0–9.0]	0.750
Intrauterine pneumonia	41				0.606
Absent		4 (10%)	3 (14%)	1 (5%)	
Present		37 (90%)	18 (86%)	19 (95%)	
Sepsis	41				0.067
Absent		32 (78%)	19 (90%)	13 (65%)	
Present		9 (22%)	2 (10%)	7 (35%)	
Intraventricular hemorrhage	41				0.130
Absent		9 (22%)	7 (33%)	2 (10%)	
Present		32 (78%)	14 (67%)	18 (90%)	
Periventricular leukomalacia	41				0.313
Absent		38 (93%)	20 (95%)	18 (90%)	
Present		3 (7%)	1 (5%)	2 (10%)	
Anemia of prematurity	41				0.002
Absent		21 (51%)	16 (76%)	5 (25%)	
Present		20 (49%)	5 (24%)	15 (75%)	
Posthemorrhagic anemia	41				0.003
Absent		34 (83%)	21 (100%)	13 (65%)	
Present		7 (17%)	0 (0%)	7 (35%)	
Respiratory distress syndrome	41				0.663
Absent		5 (12%)	2 (10%)	3 (15%)	
Present		36 (88%)	19 (90%)	17 (85%)	
Necrotizing enterocolitis	41				0.003
Absent		34 (83%)	21 (100%)	13 (65%)	
Present		7 (17%)	0 (0%)	7 (35%)	
Disseminated intravascular coagulation	41				0.004
Absent		24 (59%)	17 (81%)	7 (35%)	
Present		17 (41%)	4 (19%)	13 (65%)	
Retinopathy	41				0.140
Absent		26 (63%)	16 (76%)	10 (50%)	
Present		15 (37%)	5 (24%)	10 (50%)	

Continuous variables are expressed as median [interquartile range] and compared using the Wilcoxon rank-sum test. Categorical variables are expressed as *n* (%) and compared using Fisher's exact test. A two-sided *p*-value <0.05 was considered statistically significant. BPD—infants with confirmed bronchopulmonary dysplasia, Control—infants without bronchopulmonary dysplasia.

### 3.2 Global expression patterns and quality control

**Figure 2:**
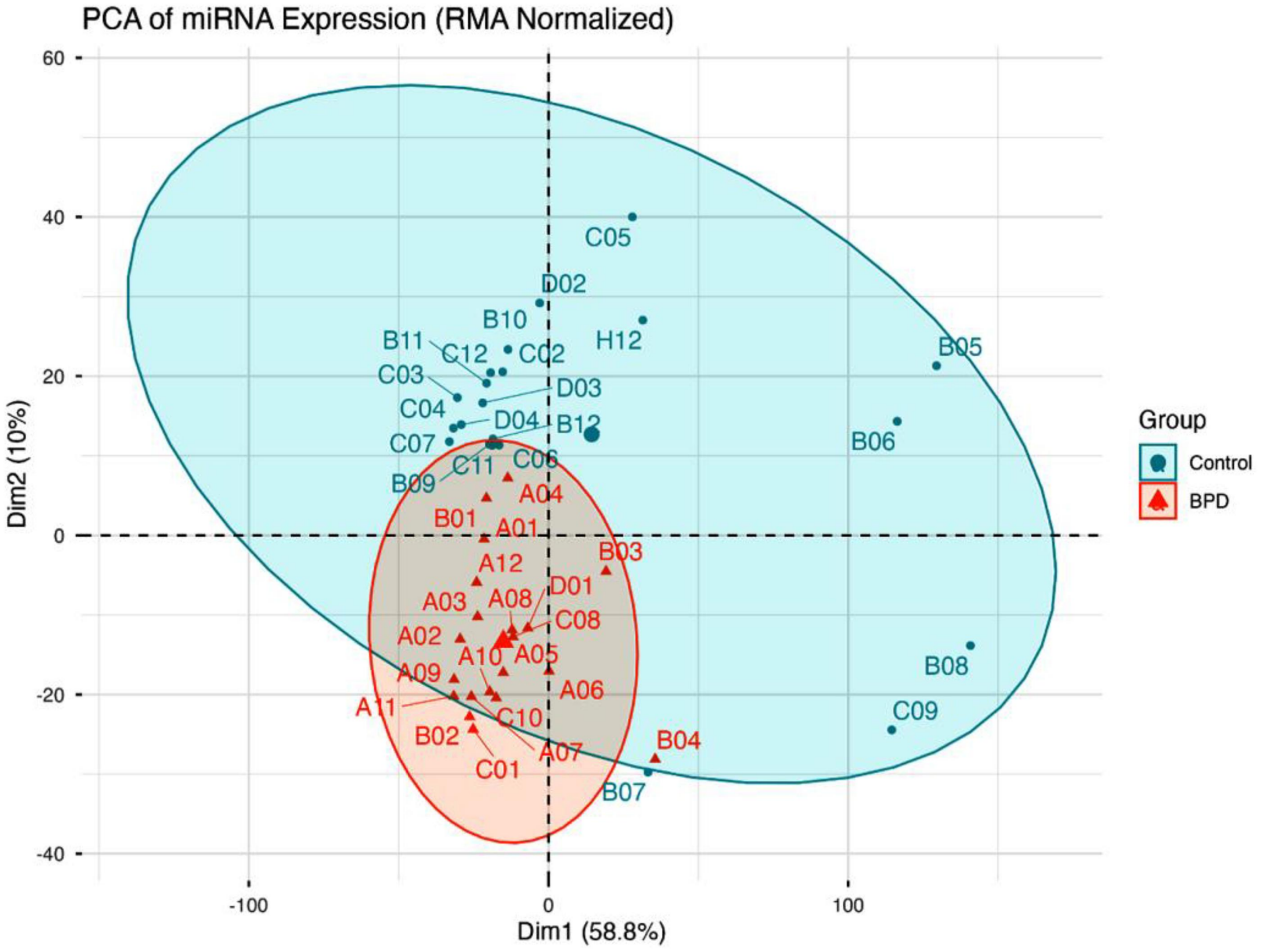
Principal component analysis (PCA) of miRNA expression profiles. Each point represents one infant, colored by group: red—BPD (*n* = 20), blue—Control (*n* = 21). Ellipses represent 95% confidence regions for each group. PCA was performed on RMA-normalized, log_2_-transformed expression values following quality filtering.

Before conducting differential expression analysis, we evaluated the quality of the normalized data and visualized the overall structure of the transcriptome profiles. Principal component analysis (PCA) revealed a tendency toward separation between BPD and control samples, with the majority of BPD + samples occupying a distinct region of the principal component space, though with partial overlap between groups (Figure 2).

### 3.3 Differential expression profiling of human miRNAs in BPD patients

**Figure 3:**
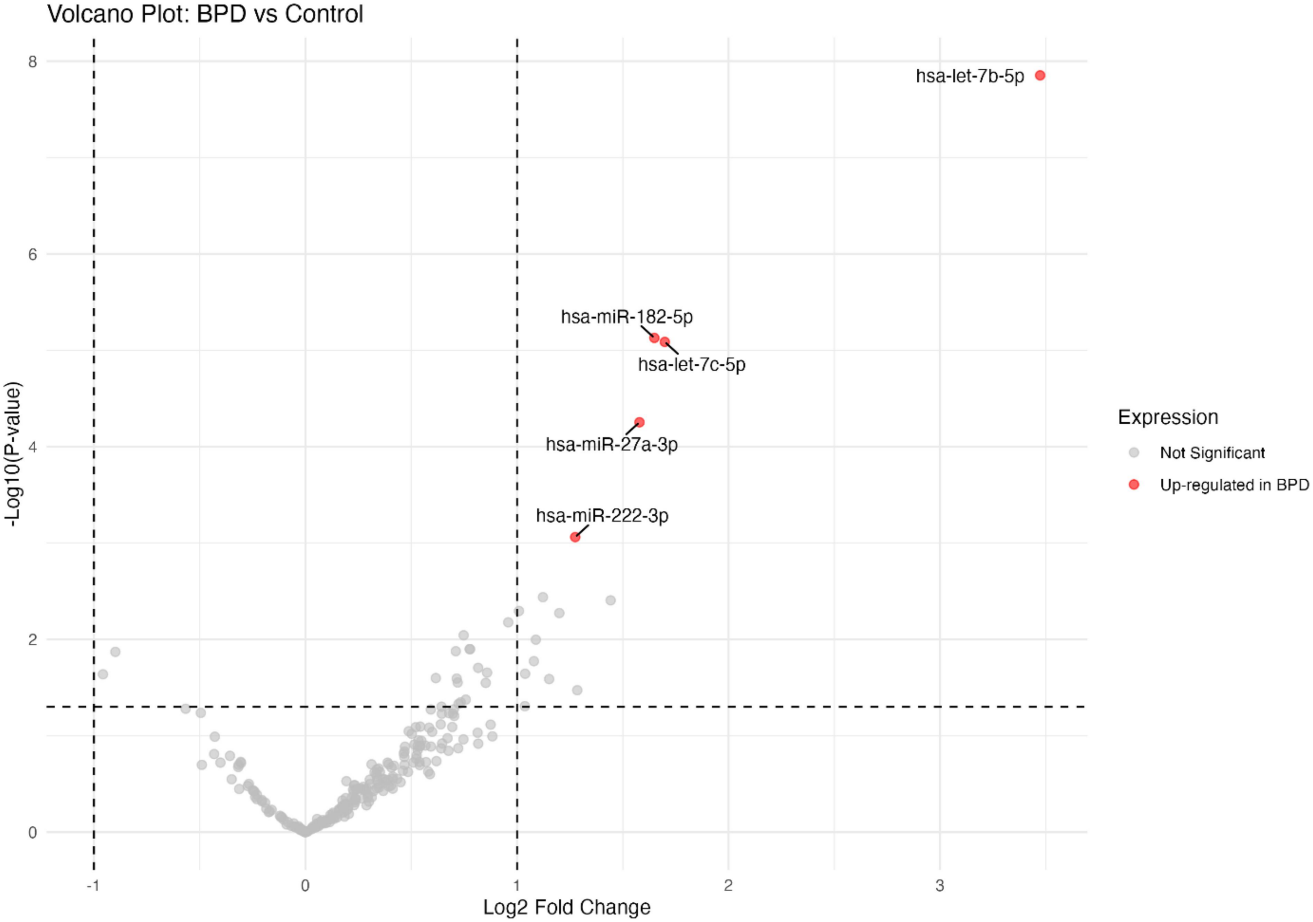
Volcano plot of differentially expressed miRNAs between BPD and control groups. The *x*-axis represents the log_2_ fold change and the *y*-axis represents the negative log_10_ of the Benjamini–Hochberg adjusted *p*-value. Each point represents one miRNA probe. Red points indicate miRNAs meeting the significance threshold (FDR < 0.05 and |log_2_FC| ≥ 1). Horizontal dashed line: FDR = 0.05; vertical dashed lines: |log_2_FC| = 1.

Primary transcriptome analysis revealed 233 miRNAs with different expression levels across the study groups (Supplementary Table S1). After a stringent statistical filtering process, including the Benjamini–Hochberg correction for multiple testing, only five molecules achieved the significance threshold (adj. *p* < 0.05), as displayed in the volcano plot (Figure 3). All five statistically significant miRNAs demonstrated a consistent pattern of upregulation in patients with confirmed bronchopulmonary dysplasia compared to controls. Detailed statistical data for these 5 miRNAs are provided in Supplementary Table S2.

The strongest signal was observed for hsa-let-7b-5p (logFC = 3.47; adj. *p* < 0.001), followed by hsa-let-7c-5p (logFC = 1.70; adj. *p* < 0.001) and hsa-miR-182-5p (logFC = 1.65; adj. *p* < 0.001) hsa-miR-27a-3p also remained significant after adjustment (logFC = 1.58; adj. *p* = 0.003), whereas hsa-miR-222-3p showed a smaller but still significant effect (logFC = 1.28; adj. *p* = 0.040).

### 3.4 Hierarchical clustering analysis

**Figure 4:**
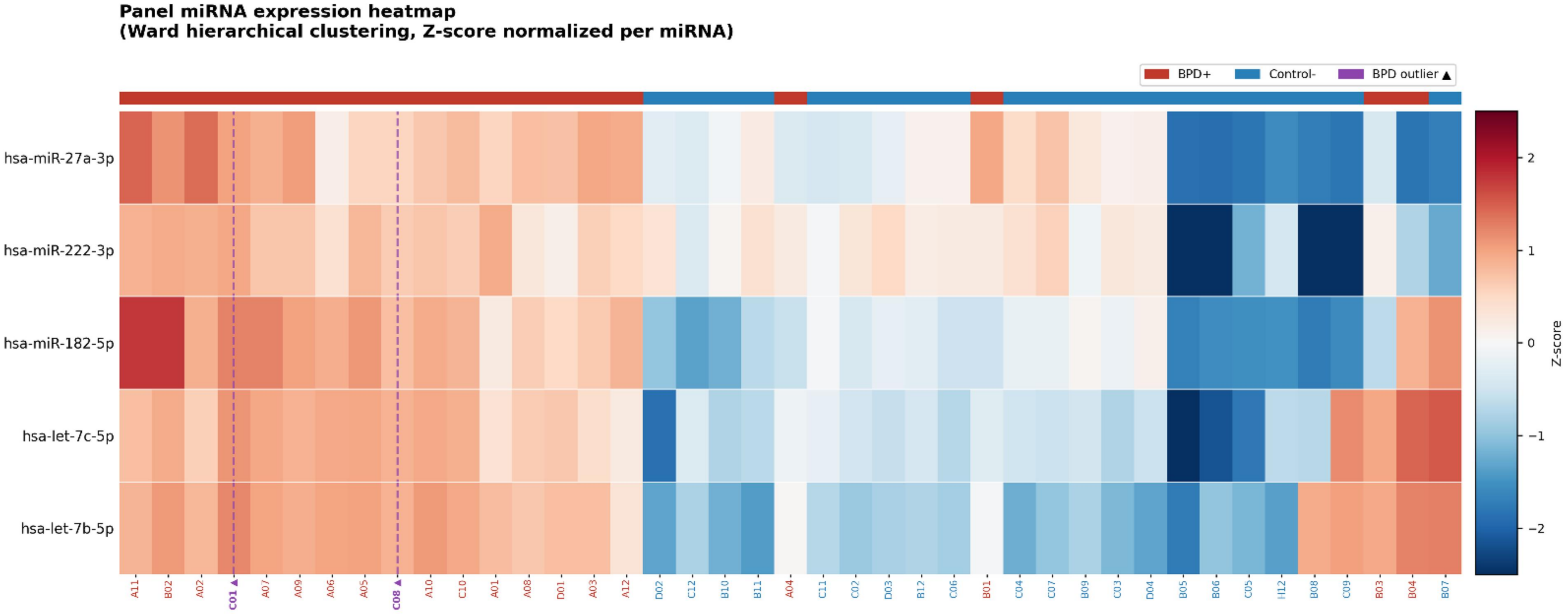
Hierarchical clustering heatmap of the five differentially expressed miRNAs. Panel miRNA expression heatmap based on hierarchical clustering with *Z*-score normalization per miRNA across all 41 samples. Rows represent the five candidate miRNAs. Columns represent individual infants. Color scale reflects *Z*-score normalized expression values: red—higher expression, blue—lower expression. Sample annotation bars at the top indicate group membership: red—BPD, blue—Control. Purple dashed lines mark BPD outlier samples that clustered with the control group.

Hierarchical clustering of the five candidate miRNAs across all 41 samples revealed an expression pattern broadly consistent with BPD status (Figure 4). The majority of BPD + samples demonstrated relative overexpression of all five miRNAs compared to controls, reflected by warmer *Z*-score values across the BPD + cluster. However, two BPD + samples clustered with the control group, suggesting biological heterogeneity within the BPD + population. The control group predominantly showed lower expression levels across all five miRNAs, with the exception of a subset of control samples in the right-hand cluster demonstrating intermediate expression values, particularly for hsa-miR-222-3p. Overall, the clustering pattern supports the directional consistency of the identified miRNA panel, while acknowledging within-group variability inherent to a heterogeneous preterm cohort.

### 3.5 Correlation analysis of miRNA expression with laboratory parameters

**Figure 5:**
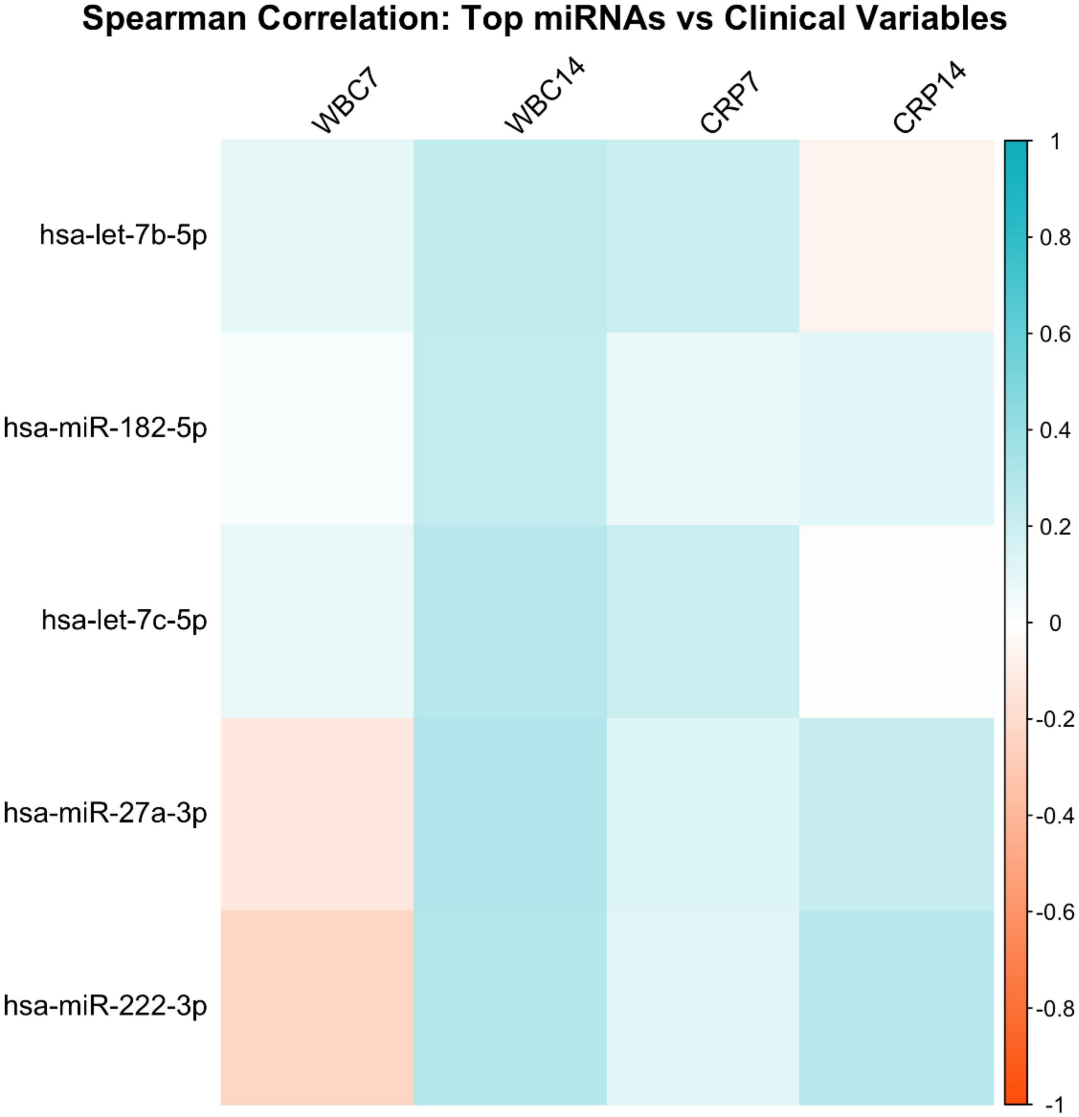
Heatmap of Spearman correlation coefficients between the five candidate miRNA expression levels and inflammatory laboratory parameters. Parameters included total white blood cell count and C-reactive protein, measured at days 7 and 14 of life. Color scale represents the Spearman correlation coefficient (*r*): red—negative correlation, blue—positive correlation.

To assess whether candidate miRNA expression reflected non-specific systemic inflammatory responses rather than BPD-specific biology, Spearman correlation analysis was performed between the expression levels of all five candidate miRNAs and inflammatory laboratory parameters, such as leukocyte count and C-reactive protein, measured at days 7 and 14 of life. No statistically significant associations were identified for any of the five miRNAs (all *p* > 0.05), indicating that their differential expression is unlikely to be driven by systemic inflammation (Figure 5). Correlation coefficients and *p*-values for all five miRNAs are provided in Supplementary Table S3. All five candidate miRNAs were therefore retained for subsequent modelling.

### 3.6 Individual diagnostic performance of candidate miRNAs

To evaluate the prognostic value of the identified differentially expressed miRNAs and their effectiveness as biomarkers for bronchopulmonary dysplasia, we performed individual ROC analysis.

Evaluation of the diagnostic significance of the identified miRNAs enabled their ranking based on the area under the ROC curve (AUC) (Table 2). The miRNA hsa-let-7c-5p demonstrated the highest discriminative ability within the study group, with an AUC of 0.91 (95% CI 0.779–1.000). Similarly, hsa-miR-182-5p (AUC = 0.89; 95% CI 0.782–0.999) and hsa-let-7b-5p (AUC = 0.89; 95% CI 0.763–1.000) showed high prognostic accuracy. The combination of markers with “very good” model quality included hsa-miR-27a-3p (AUC = 0.87, 95% CI 0.754–0.994), hsa-miR-222-3p (AUC = 0.86, 95% CI 0.740–0.970).

**Table 2:** Diagnostic performance of differentially expressed miRNAs in the study cohort.

miRNA name	AUC (95% CI)	*p*-value	Adjusted *p*-value
hsa-let-7c-5p	0.91 (0.78–1.00)	<0.001	0.001
hsa-miR-182-5p	0.89 (0.78–0.99)	<0.001	0.002
hsa-let-7b-5p	0.89 (0.76–1.00)	<0.001	0.001
hsa-miR-27a-3p	0.87 (0.75–0.99)	0.004	0.009
hsa-miR-222-3p	0.86 (0.74–0.97)	0.013	0.014

AUC values and 95% confidence intervals were computed using ROC analysis. *P*-values reflect group comparisons of miRNA expression levels. Adjusted *p*-values were obtained using Benjamini–Hochberg FDR correction. AUC, area under the curve; CI, confidence interval.

### 3.7 Results of multiple logistic regression

The next phase of statistical analysis involved stepwise logistic regression to evaluate the independent prognostic value of each of the five preselected miRNAs. Each candidate miRNA was further tested in a Firth-penalized multivariable logistic regression model adjusted for gestational age, birth weight, 5-minute Apgar score, disseminated intravascular coagulation, necrotizing enterocolitis, posthemorrhagic anemia, and anemia of prematurity. All candidate miRNAs remained significantly associated with BPD after adjustment. To account for confounding factors, each miRNA was analyzed separately alongside a set of clinical covariates that showed significance in the initial study phase. These included gestational age, birth weight, and the 5-minute Apgar score. Additionally, the analysis considered the presence of critical neonatal complications such as disseminated intravascular coagulation syndrome, necrotizing enterocolitis, posthemorrhagic anemia, and neonatal anemia. This approach helped confirm the robustness of the association between miRNA levels and bronchopulmonary dysplasia risk. Results demonstrated that the miRNAs remained statistically significant predictors at *p* < 0.05. The adjusted odds ratios, 95% confidence intervals, and *p*-values, accounting for clinical factors, are summarized in Table 3.

**Table 3:** Results of firth-penalized multivariable logistic regression analysis assessing the independent prognostic significance of each candidate miRNA for the risk of developing bronchopulmonary dysplasia.

miRNA	OR (95% CI)	*p*-value	Adjusted *p*-value
hsa-let-7b-5p	2.69 (1.49–9.28)	<0.001	<0.001
hsa-miR-222-3p	3.64 (2.47–4.88)	<0.001	<0.001
hsa-miR-182-5p	4.28 (1.82–15.52)	<0.001	<0.001
hsa-let-7c-5p	4.36 (1.80–24.01)	<0.001	<0.001
hsa-miR-27a-3p	4.47 (1.85–19.63)	<0.001	<0.001

Each miRNA was tested in a separate model adjusted for gestational age, birth weight, 5-minute Apgar score, disseminated intravascular coagulation, necrotizing enterocolitis, posthemorrhagic anemia, and anemia of prematurity. Confidence intervals are Firth profile-likelihood CIs. OR, odds ratio; CI, confidence interval.

### 3.8 Complex selection of biomarkers based on integrated scoring

**Figure 6:**
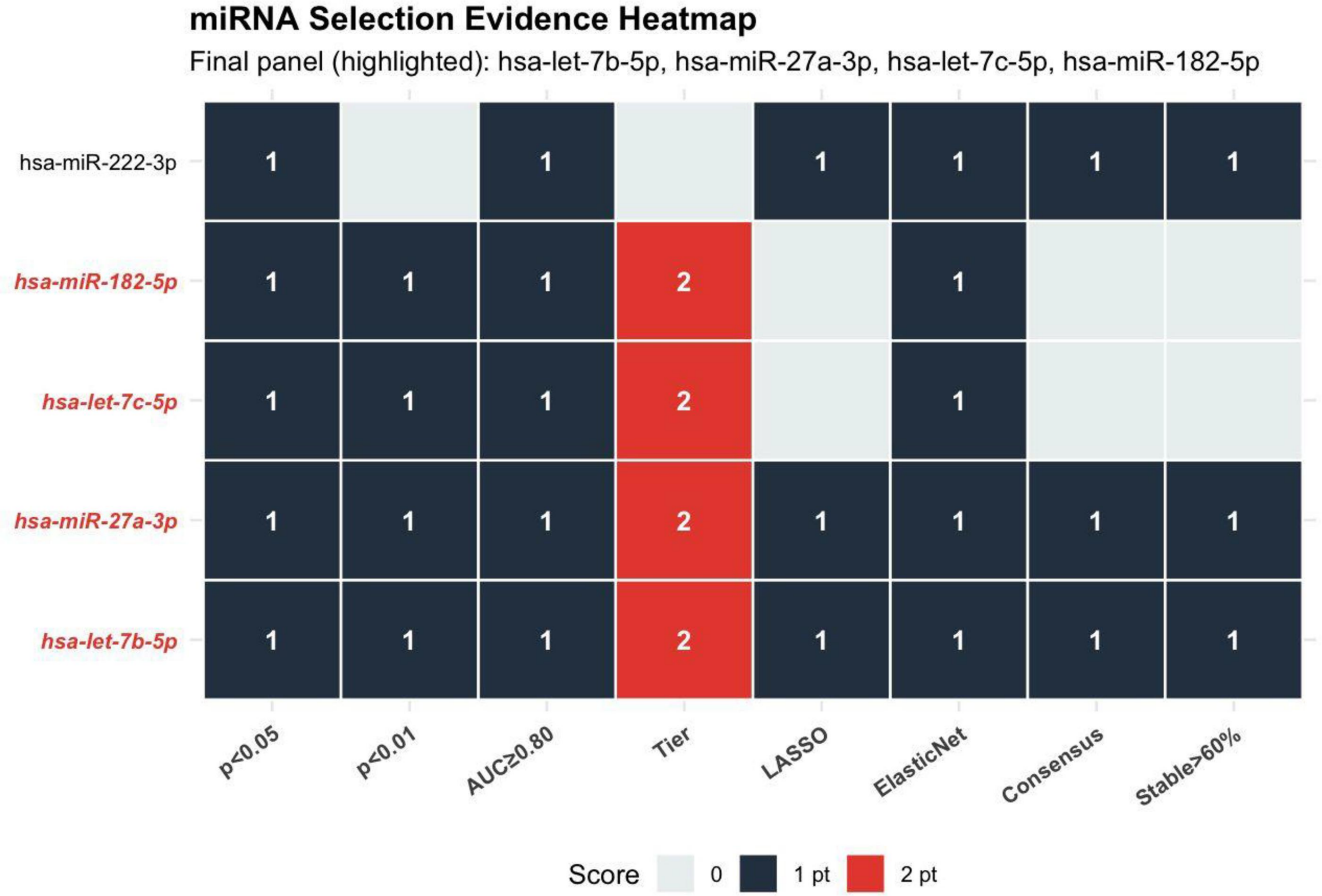
Evidence heatmap for the integrated evaluation of candidate miRNAs based on multiple performance criteria. Rows represent candidate miRNAs. Columns represent eight selection criteria: statistical significance (*p* < 0.05, *p* < 0.01), discriminative ability (AUC ≥ 0.80), tier assignment, and penalized regression stability (LASSO, ElasticNet, consensus, bootstrap frequency >60%). Cell values indicate assigned scores: 0 (light gray)—criterion not met; 1 point (dark blue)—criterion met; 2 points (red)—primary tier criterion met. miRNAs selected for the final panel are highlighted in red italic.

In the final selection stage, a multicriteria analysis was performed to identify the most promising diagnostic markers from the previously chosen candidates. The main tool for this step was the evidence heatmap in Figure 6, which integrated various performance parameters into a single analysis framework. The heatmap visualization enables simultaneous evaluation of the area under the receiver operating characteristic curve and the total score, representing each molecule's overall predictive power. The length of the graphical indicators and the coloring intensity help rank the markers based on their importance for the final model. To objectively assess the selection process, a scoring system was used. This scoring approach narrowed down the candidates to a group of miRNAs with the most consistent and reproducible performance. From this analysis, four miRNAs with the highest scores—hsa-let-7b-5p, hsa-miR-182-5p, hsa-let-7c-5p, and hsa-miR-27a-3p—were chosen for the final diagnostic panel.

### 3.9 Comparative analysis of predictive models' effectiveness

To assess the impact of the selected markers on the accuracy of bronchopulmonary dysplasia prediction, we evaluated the diagnostic performance of different computational models. This included a baseline model using solely clinical parameters, as well as combined models that incorporated clinical covariates and each of the four priority miRNAs individually. Furthermore, an integrated model combining clinical factors with all four molecular predictors was also tested.

To evaluate model reliability and estimate optimistic bias, both leave-one-out cross-validation (LOOCV) and bootstrap optimism correction (Harrell method, *B* = 500) were employed. The bootstrap-corrected AUC provides an unbiased estimate of generalization performance, while the small optimism values (0.016–0.033) confirm that regularized feature selection (LASSO + elastic net + stability selection) effectively constrained model complexity. 95% confidence intervals for all AUC values were computed using the bootstrap percentile method (*B* = 2,000 iterations).

**Figure 7:**
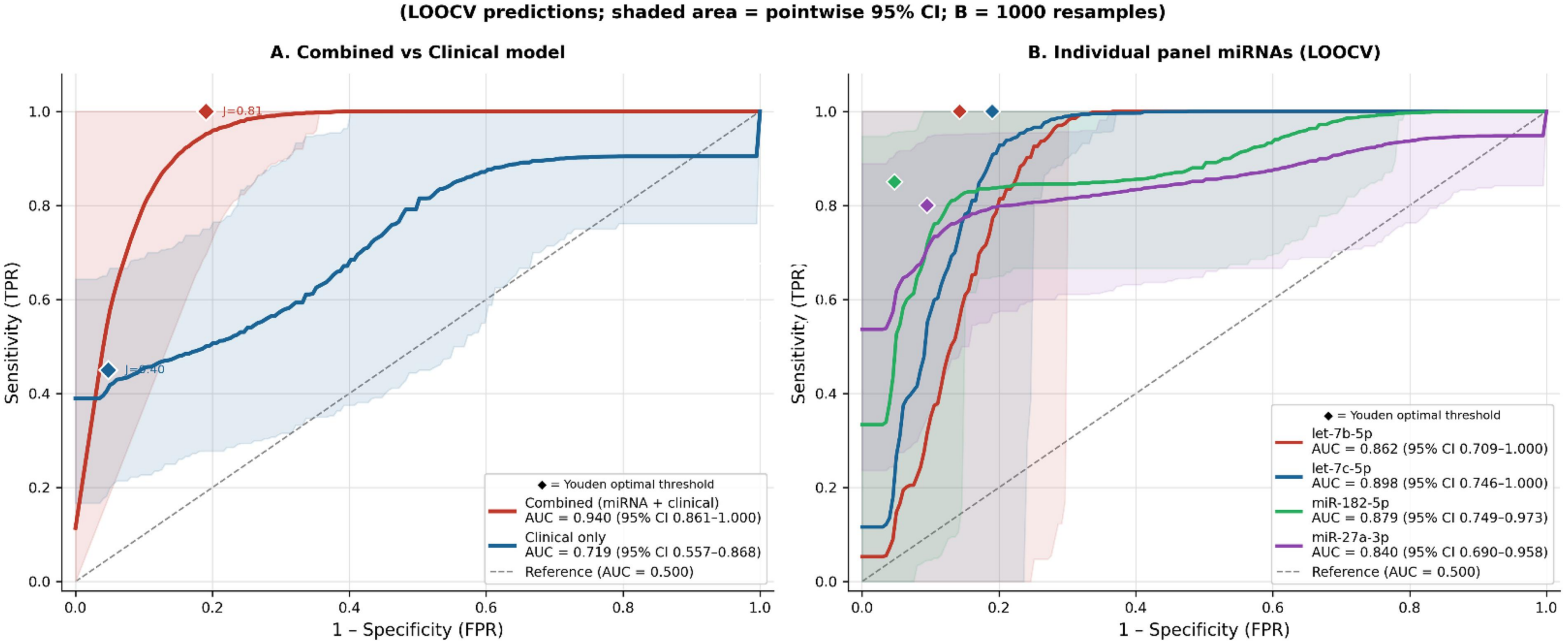
ROC curves for predictive models of bronchopulmonary dysplasia risk. **(A)** ROC curves comparing the combined four-miRNA + clinical model with the clinical-only model. **(B)** ROC curves for individual combined models for each candidate miRNA. Shaded bands represent 95% bootstrap confidence intervals (*B* = 2,000 iterations).

ROC curves for each model are presented in Figure 7 with 95% bootstrap confidence bands. The clinical-only model yielded an apparent AUC of 0.812 (bootstrap-corrected: 0.718; LOOCV AUC: 0.719, 95% CI: 0.557–0.868). The combined four-miRNA + clinical model achieved an apparent AUC of 0.995 (bootstrap-corrected: 0.941; LOOCV AUC: 0.940, 95% CI: 0.861–1.000). Bootstrap optimism was 0.033 for the clinical model and 0.016 for the combined model, indicating minimal overfitting despite the high apparent AUC. Bootstrap distribution of apparent and optimism-corrected AUC values (*B* = 2,000 iterations) for the four-miRNA LOOCV model, with 95% percentile confidence interval is observed at Supplementary Figure S1. The DeLong test confirmed superior discrimination of the combined model over clinical variables alone (*p* < 0.001). Calibration metrics for the combined model: slope = 1.050, intercept = 0.000, Brier score = 0.061, Hosmer–Lemeshow *p* = 0.159 (Supplementary Figure S2).

#### 3.9.1 Assessment of clinical utility through decision curve analysis

**Figure 8:**
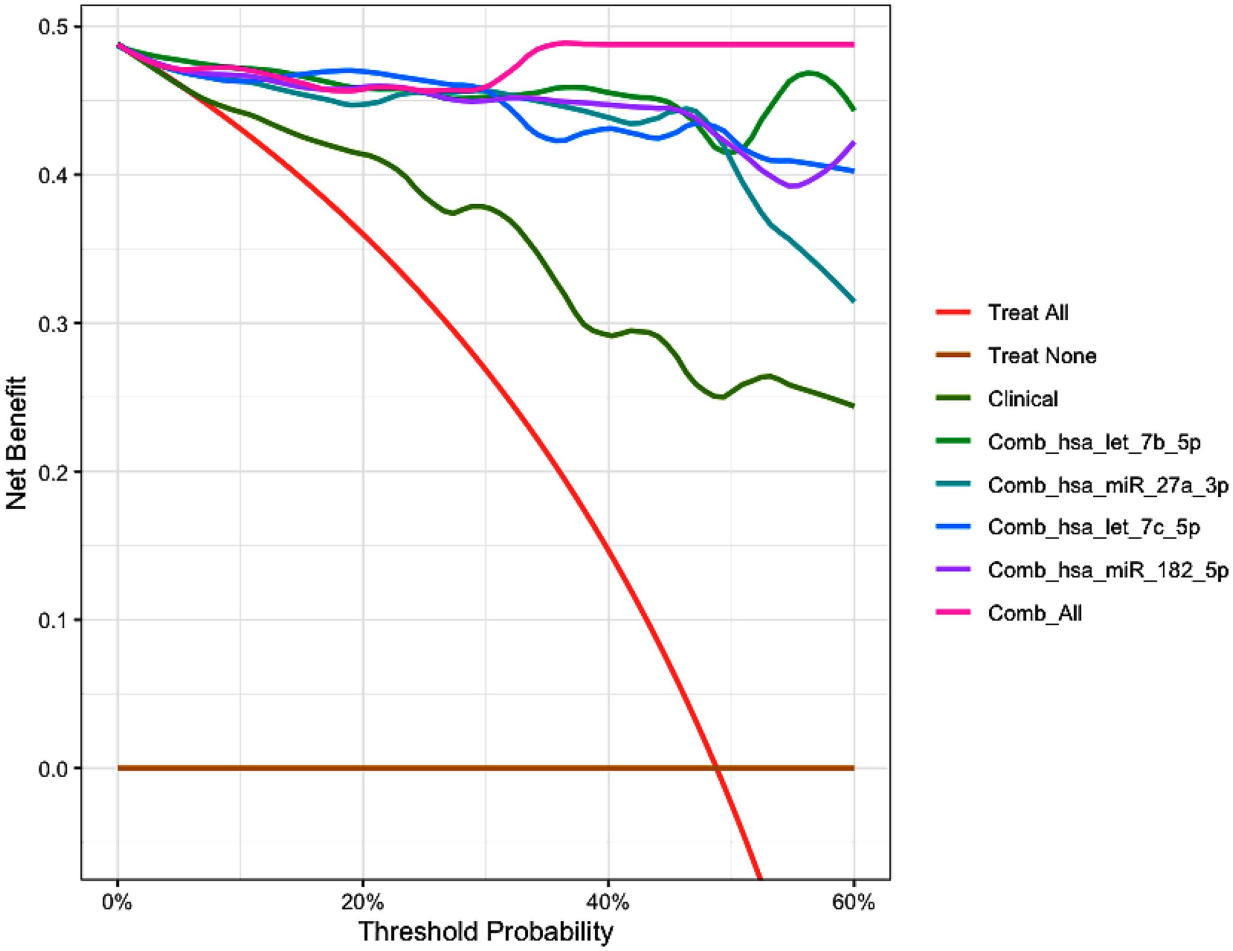
Decision curve analysis evaluating the net clinical benefit of predictive models for bronchopulmonary dysplasia across threshold probabilities of 0%–60%. The *y*-axis represents standardized net benefit. The *x*-axis represents the threshold probability. Dashed lines represent reference strategies: Treat All and Treat None.

The final assessment of the practical usefulness of the developed diagnostic algorithms was conducted through clinical decision curve analysis, with results shown in Figure 8. This approach helped measure the net benefit of the models against baseline strategies—treating all patients or no intervention. The analysis demonstrated that incorporating miRNA expression profiles offers a clear benefit over standard clinical models and empirical patient management.

Across a probability range from 10% to 60%, the combined model curves stayed above the baseline strategies of “Treat All” and “Treat None.” Notably, the advantage of models including miRNAs persisted even at low threshold probabilities.

#### 3.9.2 Functional annotation and pathway enrichment analysis of miRNA target genes

To clarify the molecular mechanisms behind the prognostic effects of hsa-let-7b-5p, hsa-let-7c-5p, hsa-miR-27a-3p, and hsa-miR-182-5p, an in silico search for their genetic targets was conducted, identifying 1,861 unique validated genes. The full list of these genes is available in Supplementary Table S4.

**Figure 9:**
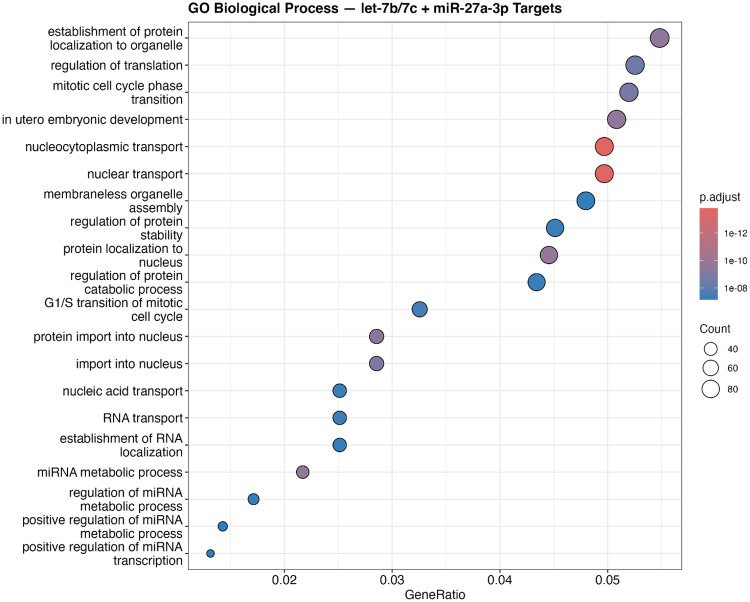
Gene ontology biological process dot plot of enriched biological processes among experimentally validated targets of the four selected miRNAs. Dot size represents the number of annotated target genes (Count). Dot color represents the Benjamini–Hochberg adjusted *p*-value (*p*.adjust). Significance threshold: *p* < 0.05 and *q* < 0.20.

Gene Ontology Biological Process analysis of experimentally validated targets of the selected miRNAs revealed significant enrichment in several biological categories (Figure 9). The highest gene ratios and most significant adjusted *p*-values were observed for processes related to intracellular protein trafficking and localization, including establishment of protein localization to organelle, protein localization to nucleus, and nucleocytoplasmic and nuclear transport. Significant enrichment was also identified for processes governing post-transcriptional regulation, including regulation of translation and miRNA metabolic processes, as well as cell cycle-related processes such as mitotic cell cycle phase transition and G1/S transition of the mitotic cell cycle. Additional significantly enriched categories included *in utero* embryonic development and membraneless organelle assembly.

**Figure 10:**
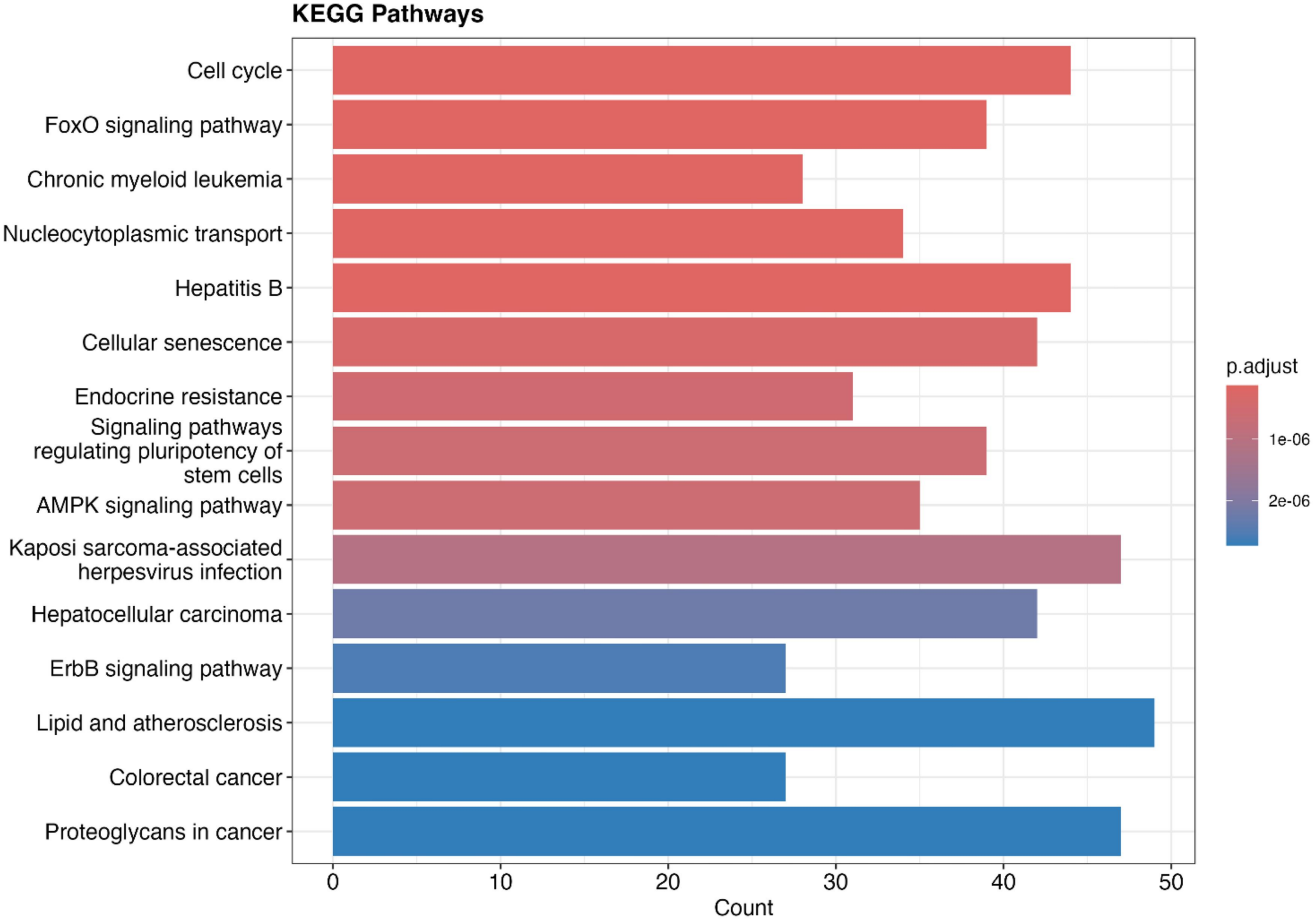
KEGG pathway enrichment bar plot for experimentally validated targets of the four selected miRNAs. Bar length represents the number of annotated target genes (Count). Bar color represents the Benjamini–Hochberg adjusted *p*-value (*p*.adjust). Significance threshold: *p* < 0.05 and *q* < 0.20.

KEGG pathway analysis of experimentally validated targets of the selected miRNAs identified 15 significantly enriched pathways, as shown in Figure 10. The most consistently enriched functional categories encompassed cell cycle regulation, including Cell cycle, Cellular senescence, and FoxO signaling pathway, as well as intracellular signaling pathways such as AMPK and ErbB signaling, and nucleocytoplasmic transport. Several additional pathways related to oncological and viral contexts were also enriched, likely reflecting the broad regulatory scope of the selected miRNAs rather than direct biological relevance to BPD.

Collectively, functional enrichment analysis identified cell cycle regulation, intracellular signaling, and nucleocytoplasmic transport as the predominant biological processes and pathways represented among the validated targets of the selected miRNAs.

## 4 Discussion

Prediction of bronchopulmonary dysplasia remains a significant challenge in neonatology due to the disease's complex pathogenesis. This study identified and validated a panel of four miRNAs—hsa-let-7b-5p, hsa-let-7c-5p, hsa-miR-27a-3p, and hsa-miR-182-5p—that exhibit high predictive value for BPD development in very low birth weight preterm infants. Whole blood was selected as the biomaterial, providing several advantages over tracheal aspirate in very low- and extremely low-birth-weight preterm infants (31). While blood serves as a systemic indicator of general homeostatic changes, it enables less invasive monitoring and reflects a broad spectrum of circulating regulators (12, 32–34).

The added clinical value of the miRNA-based model was evaluated by comparing its discriminatory performance with that of a baseline model incorporating standard clinical parameters. The clinical-only model produced a bootstrap-corrected AUC of 0.718 and LOOCV AUC 0.719, indicating modest predictive accuracy for conventional neonatal parameters. Incorporation of the four-miRNA panel with clinical predictors substantially improved discrimination, resulting in a bootstrap-corrected AUC of 0.941 and LOOCV AUC 0.940. The DeLong test demonstrated that this improvement was statistically significant (*p* < 0.001). Bootstrap optimism was minimal for both models, indicating that regularized feature selection effectively limited model complexity. These results indicate that circulating miRNAs may capture pathogenic signals of early lung injury that are not reflected in standard clinical variables. However, due to the small cohort size and lack of external validation, these findings should be considered preliminary.

The potential clinical applicability of the proposed approach was assessed using decision curve analysis. Across threshold probabilities ranging from 10% to 60%, the combined model consistently yielded higher net benefit than both the clinical-only model and default management strategies. The clear decision advantage at low threshold probabilities is particularly important in the neonatal context, where the risk of missing a high-risk infant far outweighs the burden of unnecessary intervention. Determining whether this benefit leads to tangible clinical impact requires prospective validation.

A central analytical challenge in this study was disentangling BPD-specific miRNA dysregulation from signals attributable to extreme prematurity and its associated morbidities. The cohort exhibited pronounced baseline imbalances in gestational age, birth weight, and the prevalence of severe complications, including necrotizing enterocolitis and posthaemorrhagic anemia—all of which plausibly influence circulating miRNA profiles independent of BPD status. Establishing that the identified associations were not simply proxies for these confounders was therefore a methodological prerequisite rather than an optional sensitivity check.

Firth-penalized multivariable logistic regression was selected for this purpose, given its established suitability for small, imbalanced samples where standard maximum likelihood estimation produces biased coefficients. After simultaneous adjustment for gestational age, birth weight, 5-minute Apgar score, and the principal neonatal comorbidities, all four candidate miRNAs retained statistically significant independent associations with BPD. This pattern of sustained significance across a broad set of covariates suggests that miRNA dysregulation at days 10–14 of life captures molecular processes related to BPD pathogenesis rather than reflecting constitutional immaturity alone. That said, residual confounding cannot be excluded in a cohort of 41 infants, and these associations should be regarded as hypothesis-generating pending replication in larger independent samples.

The biological specificity of the panel was further interrogated through correlation-based sensitivity analysis. Given that systemic inflammation is both a recognized driver of BPD and a potent modulator of circulating miRNA levels, any association between candidate miRNAs and markers such as white blood cell count or C-reactive protein would raise concern that the signal reflects non-specific inflammatory activation rather than disease-specific biology. Spearman correlation analysis between all five candidate miRNAs and WBC and CRP measured at days 7 and 14 of life revealed no statistically significant associations at either time point. While the absence of correlation does not exclude more subtle inflammatory confounding, these findings are at least consistent with the interpretation that the differential expression of the identified miRNAs is not primarily driven by generalized inflammatory activity.

The final diagnostic panel was constructed by deliberately prioritizing cross-method consistency over raw performance ranking. Instead of selecting candidates based on a single metric, which would be particularly susceptible to sampling instability in a cohort of this size, the scoring framework required concordant evidence across statistically and algorithmically independent criteria. Molecules that ranked highly on one dimension but did not replicate that performance across others were systematically deprioritized. The behavior of hsa-miR-222-3p illustrates this approach. Although it demonstrated strong individual discriminative ability, its inclusion did not provide additional predictive value when the remaining four candidates were considered together. This pattern suggests shared regulatory targeting rather than an independent biological signal. Consequently, the composition of the final panel reflects analytical robustness rather than optimization for any single performance criterion.

To ensure the biological validity of the mathematically selected panel and minimize false-positive results, the functional analysis was restricted to 1,861 consensus, experimentally validated target genes obtained from high-confidence repositories. Functional enrichment analysis of these verified targets elucidated the molecular context underlying the predictive model. There was a significant predominance of genes involved in cell cycle regulation, cellular senescence, intracellular protein trafficking, and stress signaling cascades, including the FoxO and AMPK pathways (35, 36), supporting the signature's pathophysiological association with lung injury mechanisms. These mechanisms align with the established pathogenesis of lung injury in preterm infants, in which chronic inflammation, oxidative stress, and impaired epithelial regeneration contribute to the development of BPD (37, 38).

The identified miRNAs form a coordinated regulatory network. Thus, let-7b-5p and let-7c-5p are involved in the control of cell cycle transitions and senescence induction (39–41), which may contribute to the accumulation of functionally inactive cells in lung tissue. In turn, miR-27a-3p is associated with the activation of inflammatory cascades (42, 43), and miR-182-5p regulates the balance between apoptosis and proliferation (44–46). The combined action of these miRNAs reflects key mechanisms of lung injury, such as impaired cell renewal, increased inflammation, and decreased regenerative capacity (47).

The apparent discrepancy between the elevated let-7c-5p expression observed in our cohort and experimental evidence suggesting a protective role for this molecule in lung homeostasis (48) warrants careful consideration. Rather than representing a straightforward contradiction, this inconsistency most plausibly reflects the context-dependence of miRNA function, whereby the regulatory outcome of a given molecule is shaped by the developmental stage of the target tissue, the composition of co-expressed transcripts, and the broader signaling environment at the time of sampling. Concordant elevations reported in independent clinical cohorts (49) further suggest that the direction of let-7c-5p dysregulation observed here is not an isolated finding, though its mechanistic interpretation remains open.

These discrepancies highlight a broader methodological challenge in the field. The expression of circulating miRNAs is sensitive to postnatal age at sampling, the choice of biological compartment, and platform-specific detection characteristics, each of which introduces systematic variability that complicates cross-study comparison in the absence of harmonized protocols (50). Until prospective multicentre studies with standardized sampling procedures and unified analytical pipelines are available, the prognostic boundaries of let-7 family miRNAs in the neonatal clinical setting will remain incompletely defined. The methodological framework applied in the present study was designed with this objective in mind, and the reported findings are intended to contribute a reproducible reference point toward the development of such protocols.

### 4.1 Limitations of the study

This study has several limitations that should be considered when interpreting the findings. The cohort size of 41 infants, while adequate for an exploratory analysis, limits statistical power and generalisability to the broader population of VLBW and ELBW preterm infants. Although the significant between-group differences in gestational age and birth weight were addressed through covariate adjustment in both the limma differential expression model and Firth-penalized logistic regression, the possibility that residual confounding influenced the miRNA expression profiles cannot be entirely excluded in a sample of this size.

RNA quality assessment represented a further methodological constraint. Integrity was evaluated by UV spectrophotometry, fluorometric quantification, and conventional agarose gel electrophoresis. However, capillary electrophoresis platforms such as the Agilent Bioanalyzer or TapeStation, which provide a quantitative RNA integrity number and resolve the small-RNA fraction with greater sensitivity, were not employed. Conventional gel electrophoresis is less discriminating for detecting partial degradation of low-molecular-weight species and subtle inter-sample differences in small RNA integrity. Therefore, it cannot be fully excluded. This consideration underscores the need for independent RT-qPCR confirmation, which remains the reference standard for miRNA quantification and is a primary objective of the planned follow-up study using the same patient samples.

Although bootstrap optimism correction was applied and the resulting optimism values were small, miRNA candidate selection was performed on the full dataset prior to cross-validation, which may still introduce a residual degree of optimistic bias into the reported performance estimates. The in silico target gene analysis, while deliberately restricted to experimentally validated interactions, remains inferential, and the functional role of the identified miRNAs in BPD pathogenesis requires confirmation through mechanistic studies examining target protein expression in relevant biological systems.

Several clinical variables potentially relevant to BPD risk, including cumulative FiO_2_ exposure, total mechanical ventilation duration, and post-sampling transfusion data, were not uniformly available across all patients and could not be incorporated into the predictive models. The study applied the 2018 NIH consensus diagnostic criteria for BPD. The Jensen 2019 severity-graded classification, which is increasingly adopted in contemporary literature, will be implemented in the expanded follow-up cohort.

Finally, the single-center design and the absence of an external validation cohort mean that the present findings should be regarded as discovery-phase results. The robustness and generalisability of the proposed model require confirmation in independent, prospectively recruited populations across multiple centers before any consideration of clinical translation.

## 5 Conclusion

This discovery-phase study demonstrates that integrating miRNA expression profiles with clinical data yields a promising model for early BPD risk assessment in VLBW and ELBW preterm infants, achieving an LOOCV AUC of 0.940 in internal validation. Covariate-adjusted differential expression analysis identified five candidate miRNAs whose expression was independent of gestational age, birth weight, and systemic inflammatory parameters, supporting the biological plausibility of the identified panel. A multi-criterion evidence scoring approach enabled the selection of four miRNAs, comprising hsa-let-7b-5p, hsa-miR-27a-3p, hsa-let-7c-5p, and hsa-miR-182-5p, whose integration with clinical predictors substantially improved discriminative performance compared to a clinical-only model. These findings represent a preliminary but methodologically rigorous step toward molecular risk stratification of BPD in the early neonatal period. Nevertheless, the results should be interpreted within the constraints of a single-centre exploratory cohort, and independent external validation alongside RT-qPCR confirmation of the microarray findings are necessary prerequisites before the proposed approach can be considered for clinical application.

## Data Availability

The data associated with this study has been deposited in the NCBI Gene Expression Omnibus (GEO) under accession number GSE336376.
