# Supplementary material for: Integration of miRNA profiles and clinical data for early risk assessment of bronchopulmonary dysplasia in VLBW and ELBW newborn infants: a discovery study
Source: Front Pediatr. 2026 Jul 6;14:1853322. doi: 10.3389/fped.2026.1853322 (PMC13381776; doi:10.3389/fped.2026.1853322)
Supplement: Supplementary file 1 [file Datasheet1.zip › Supplementary files Revised/Table S2 revised.docx]

**Table S2. Differential expression statistics for the five miRNAs meeting the significance threshold (FDR < 0.05 and |log₂FC| ≥ 1) in the covariate-adjusted limma analysis. logFC — log₂ fold change (BPD vs. control); Average expression — mean log₂-transformed RMA-normalised intensity across all samples; t — moderated t-statistic; p-value — nominal p-value from empirical Bayes moderation; Adjusted p-value — Benjamini–Hochberg FDR-corrected p-value; B-statistic — log-odds of differential expression. All five miRNAs were upregulated in infants who developed BPD relative to controls.**

| **miRNA**  **name** | **logFC** | **Average expression** | **t** | **p-value** | **Adjusted**  **p-value** | **B-statistic** | **Expression** |
| --- | --- | --- | --- | --- | --- | --- | --- |
| hsa-let-7b-5p | 3.47 | 8.43 | 6.86 | <0.001 | <0.001 | 9.53 | Up-regulated in BPD |
| hsa-let-7c-5p | 1.70 | 8.46 | 5.01 | <0.001 | <0.001 | 3.35 | Up-regulated in BPD |
| hsa-miR-182-5p | 1.65 | 5.72 | 5.04 | <0.001 | <0.001 | 3.44 | Up-regulated in BPD |
| hsa-miR-27a-3p | 1.58 | 5.77 | 4.43 | <0.001 | 0.003 | 1.50 | Up-regulated in BPD |
| hsa-miR-222-3p | 1.28 | 6.64 | 3.56 | <0.001 | 0.040 | -1.11 | Up-regulated in BPD |
