# Supplementary material for: Integration of miRNA profiles and clinical data for early risk assessment of bronchopulmonary dysplasia in VLBW and ELBW newborn infants: a discovery study
Source: Front Pediatr. 2026 Jul 6;14:1853322. doi: 10.3389/fped.2026.1853322 (PMC13381776; doi:10.3389/fped.2026.1853322)
Supplement: Supplementary file 1 [file Datasheet1.zip › Supplementary files Revised/Table S3 revised.docx]

**Table S3. Spearman correlation analysis between the expression levels of five candidate miRNAs and systemic inflammatory parameters measured at days 7 and 14 of life. r — Spearman correlation coefficient. No statistically significant associations were identified for any miRNA at either time point (all p > 0.05), supporting the interpretation that differential miRNA expression is not primarily driven by generalised inflammatory activity. WBC — white blood cell count (×10⁹/L); CRP — C-reactive protein (mg/L).**

| **miRNA** | **WBC**  **(day 7, r)** | **WBC**  **(day 7, p-value)** | **WBC**  **(day 14, r)** | **WBC**  **(day 14, p-value)** | **CRP**  **(day 7, r)** | **CRP**  **(day 7, p-value)** | **CRP**  **(day 14, r)** | **CRP**  **(day 14, p-value)** |
| --- | --- | --- | --- | --- | --- | --- | --- | --- |
| hsa-let-7b-5p | 0.09 | 0.56 | 0.25 | 0.12 | 0.21 | 0.19 | -0.07 | 0.68 |
| hsa-let-7c-5p | 0.08 | 0.64 | 0.28 | 0.08 | 0.21 | 0.19 | -0.02 | 0.91 |
| hsa-miR-182-5p | 0.04 | 0.82 | 0.24 | 0.13 | 0.08 | 0.63 | 0.12 | 0.46 |
| hsa-miR-27a-3p | -0.12 | 0.44 | 0.31 | 0.05 | 0.12 | 0.45 | 0.22 | 0.18 |
| hsa-miR-222-3p | -0.22 | 0.17 | 0.29 | 0.07 | 0.12 | 0.46 | 0.28 | 0.08 |
