# Supplementary figures and images for: Integration of miRNA profiles and clinical data for early risk assessment of bronchopulmonary dysplasia in VLBW and ELBW newborn infants: a discovery study
Source: Front Pediatr. 2026 Jul 6;14:1853322. doi: 10.3389/fped.2026.1853322 (PMC13381776; doi:10.3389/fped.2026.1853322)

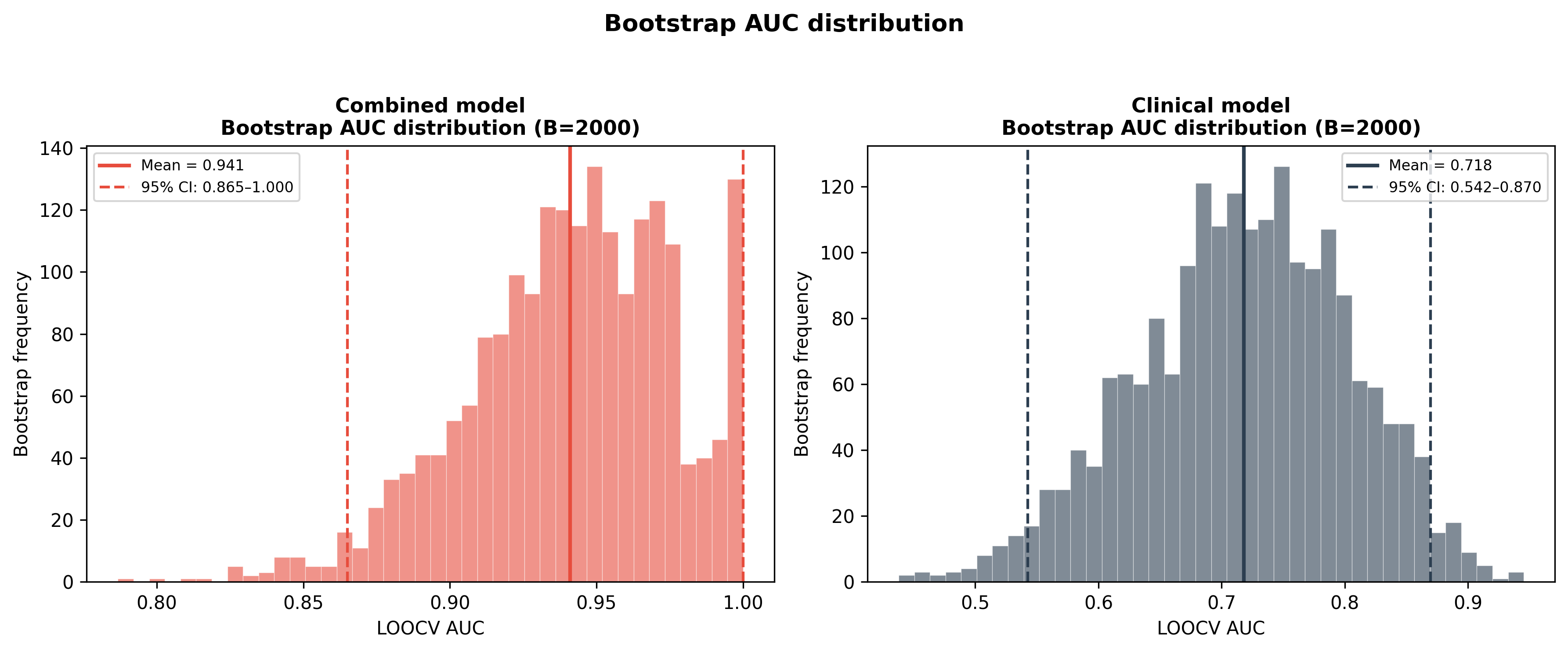

Supplement: Supplementary file 1 [file Datasheet1.zip › Supplementary files Revised/Figure S1.png]

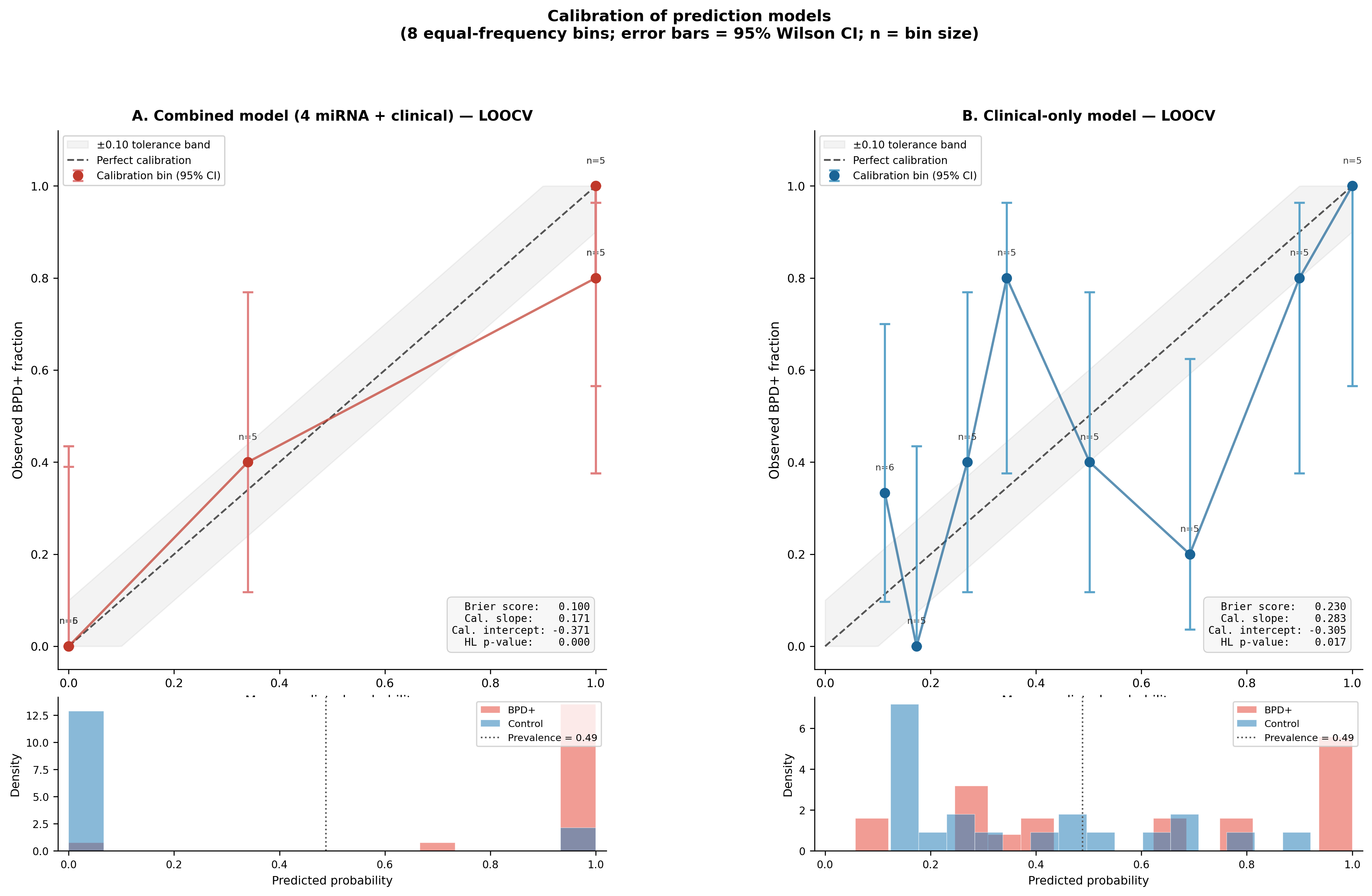

Supplement: Supplementary file 1 [file Datasheet1.zip › Supplementary files Revised/Figure S2.png]
